# Supplementary material for: Multifaceted Functional Liposomes: Theranostic Potential of Liposomal Indocyanine Green and Doxorubicin for Enhanced Anticancer Efficacy and Imaging
Source: Pharmaceutics. 2025 Mar 7;17(3):344. doi: 10.3390/pharmaceutics17030344 (PMC11944616; doi:10.3390/pharmaceutics17030344)
Supplement: Supplementary file 1 [file pharmaceutics-17-00344-s001.zip › pharmaceutics-3468022-supplementary.pdf]

Supplementary Materials

Multifaceted Functional Liposomes: Theranostic Potential of Liposomal Indocyanine Green and Doxorubicin for Enhanced Anticancer Efficacy and Imaging

Wei-Ting Liao [1,2], Dao-Ming Chang [3], Meng-Xian Lin [1], Te-Sen Chou [1], Yi-Chung Tung [3*], and Jong-Kai Hsiao [1,2*]

[1] Department of Medical Imaging, Taipei Tzu Chi General Hospital, Buddhist Tzu-Chi Medical Foundation, New Taipei City 23142, Taiwan
[2] School of Medicine, Tzu Chi University, Hualien 97004, Taiwan
[3] Research Center for Applied Sciences, Academia Sinica, Taipei 11529, Taiwan
* Correspondence: YC Tung: tungy@gate.sinica.edu.tw; Tel: +886-2-2787-3138; JK Hsiao: Jongkai@tzuchi.com.tw; Tel: +886-2-66289779 ext 61714

Table S1. The detailed liposome drug concentrations used in different in vitro and in vivo experiments.

**Initial Concentration:**<br>Lipo-ICG/DOX: ICG 896.8 μg/mL, DOX 688.8 μg/mL
<u>Cellular uptake</u><br>1. control<br>2. ICG 62 μg/mL, DOX 50 μg/mL<br>3. ICG 31 μg/mL, DOX 25 μg/mL<br><br><u>Thermalgram images</u><br>ICG 896.8 μg/mL, DOX 688.8 μg/mL<br><br><u>Cell viability</u><br>1. ICG 1.25 μg/mL, DOX 62.5 nM<br>2. ICG 2.5 μg/mL, DOX 125 nM<br>3. ICG 5 μg/mL, DOX 250 nM<br>4. ICG 10 μg/mL, DOX 500 nM<br>5. ICG 10 μg/mL<br><br><u>RNA-seq</u><br>1. control<br>2. ICG 50 μg/mL, DOX 38.4 μg/mL<br>3. ICG 50 μg/mL, DOX 38.4 μg/mL, +light 20 min
**Initial Concentration:**<br>Lipo-ICG/DOX: ICG 651.1 μg/mL, DOX 520.4 μg/mL
<u>Animal experiments</u><br>1. Control<br>2. ICG 7.5 mg/kg, DOX 6 mg/kg<br>(ICG 150.1 μg/mL, DOX 119.9 μg/mL)<br>3. ICG 7.5 mg/kg, DOX 6 mg/kg, +light 15 min<br>(ICG 150.1 μg/mL, DOX 119.9 μg/mL)

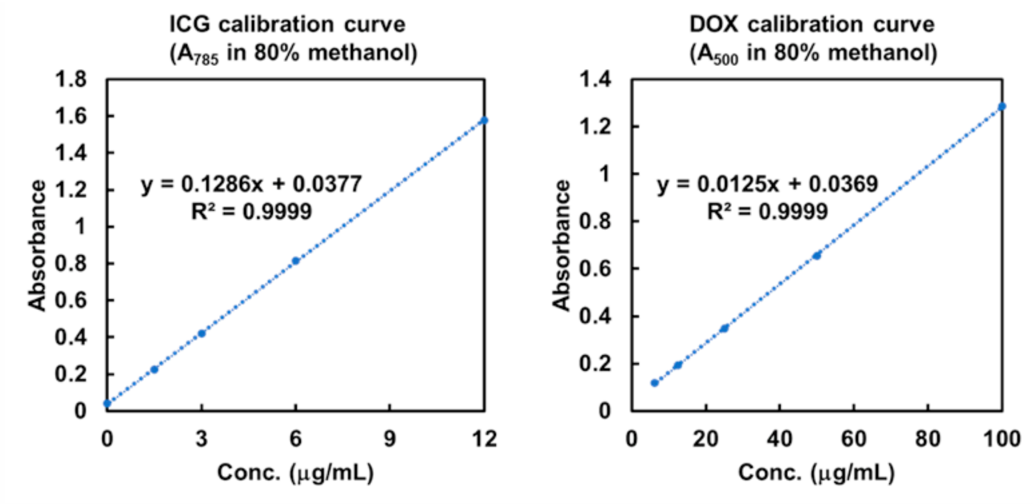

Figure S1. The calibration curves of ICG and doxorubicin established based on the absorbance measurement for the concentration estimation.

In vitro cell viability analysis of free drugs

To evaluate free drug efficacy, in vitro cell experiments were conducted using the A549 lung cancer cell line. The cells derived from the same batch were divided into seven populations and treated with liposomes at various concentrations (ICG: 0 to 100 μg/mL, doxorubicin: 0 to 500 nM). The A549 cells were cultured in 96-well plates with 2.5×10^4 cells per well, and incubated in a cell culture incubator maintained at 37°C with 5% CO_2 for 1 to 3 days, and irradiate with 780 nm for 30 minutes at day 2. Following the treatment, the medium containing liposomes was removed, and the cells were washed with DPBS before adding DMEM supplemented with the PrestoBlue™ Cell Viability Reagent (Invitrogen A13262, ThermoFisher Scientific) to estimate cell viability for drug efficacy quantification. Fluorescence intensity was measured at an excitation/emission wavelength of 560/590 nm using a multi-mode microplate reader (BioTek Synergy 2, ThermoFisher Scientific) in the bottom-read mode.

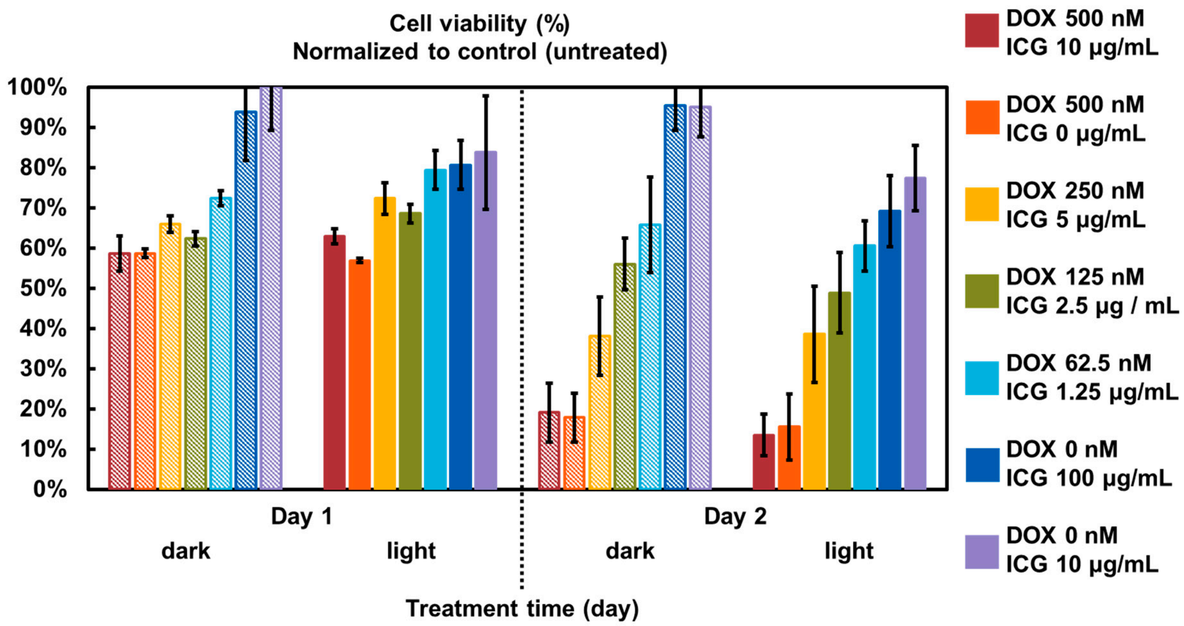

Figure S2. The cell viability of A549 cells treated with combinations of free ICG and doxorubicin for 24 and 28 hours without and with the light exposure.
